# COVID-19-associated leukocytoclastic vasculitis leading to gangrene and amputation

**DOI:** 10.1016/j.idcr.2021.e01117

**Published:** 2021-04-06

**Authors:** Khalid Omar Alattar, Farah Noaman Subhi, Ayesha Humaid Saif Alshamsi, Nadereh Eisa, Niaz Ahmed Shaikh, Jehangir Afzal Mobushar, Asma Al Qasmi

**Affiliations:** Rashid Hospital, Internal Medicine Department, Umm Hurair 2, Dubai, United Arab Emirates

**Keywords:** COVID-19, Vasculitis, Gangrene, Amputation, Pneumonia

## Abstract

A 41-year-old male with type 2 diabetes mellitus (T2DM) presented with complaints of recent onset limb weakness, diffuse body rash and fever. Computerized Tomography (CT) scan of the brain did not reveal a stroke but laboratory investigations of the patient portrayed multi-systemic involvement. Naso-pharyngeal swab for severe acute respiratory syndrome coronavirus 2 (SARS-CoV-2) was taken which resulted as positive. Soon after, a biopsy of the skin lesions revealed histo-pathological features of leukocytoclastic vasculitis. The patient was further investigated for connective tissue disease and vasculitis only to yield a negative result for all relevant antibodies, with the exception of the anti-phospholipid antibody which was positive. The patient suffered through a complex and prolonged hospital stay that required the input of multiple sub-speciality teams. Although initially presenting with a normal chest X-ray the patient went on to have severe bilateral pneumonia and a progression of initial skin rash leading to severe necrosis of the left foot with dry gangrene of the left big toe. Due to these issues, coronavirus-disease-2019 (COVID-19) aimed therapy was started along with multiple skin debridements, antibiotics and eventual amputation of the patient’s affected large toe. The following case-study details all the before-mentioned events with discussion of the possible reasons behind the patient’s presentation and eventual outcome.

## Introduction

The world has been overwhelmed and truly tested in respect to its ability to contain the novel coronavirus since its outbreak was first reported on the 31st of December 2019. The person-to-person spread of this positive-sense single-stranded ribonucleic acid (RNA) virus triggered declaration of a global pandemic on March 11th 2020 [1]. Although thought to affect the respiratory system primarily, a multitude of research including this case report demonstrates systemic involvement and atypical presentations. Still, regardless of the many organ systems being addressed, skin manifestations are focused on, with few prior articles demonstrating such severe lesions, especially in the Middle-East [2].

## Case presentation

A 41 year-old male known case of T2DM presented to the emergency department complaining of left upper limb weakness for two hours. This was associated with fever and rhinorrhea for two days prior and a body rash. The rash appeared two weeks before the limb weakness and was diffuse, non-pruritic, non-painful and initially red but now black in color in certain areas. The lesions first appeared on both distal lower limbs then went on to spread to both upper limbs and the trunk.

It was the first time the patient experienced such symptoms and he denied any sensory disturbances, slurring of speech, shortness of breath, cough, sore throat, headache and pain. Joint pain, oral ulcers, dry eyes and mouth were also denied. The patient inconsistently used an unknown oral anti-diabetic medication for many years, did not take any new medications recently and had no history of prior diabetic complications. No known allergies or family history of similar issues were reported and there was no recent travel history or possibly-ill close contacts to the patient.

**Fig. 1:**
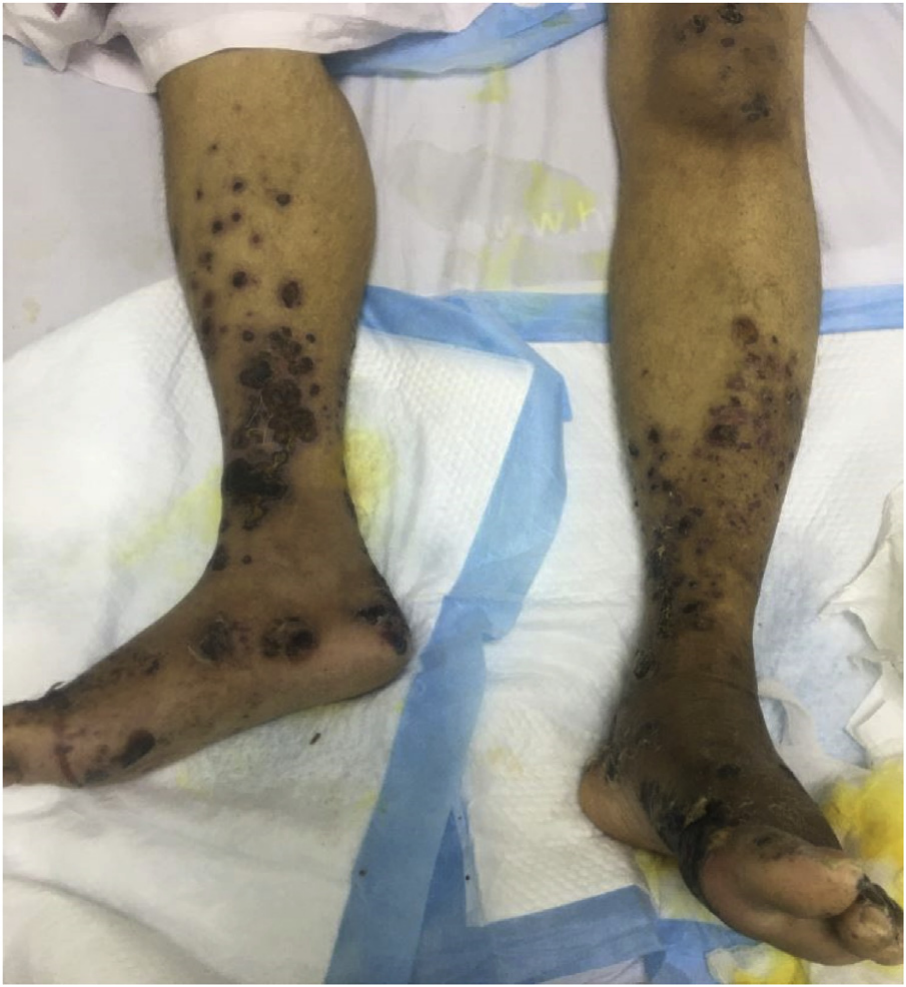
Skin Lesions of the Lower Limbs. Bilateral lower limbs with diffuse islands of necrosis, superficial ulcerations and crusting spanning up to the lower knees. Left foot can be seen with the largest necrotic patch of approximately 8 × 6 cms over the medial plantar area.

**Fig. 2:**
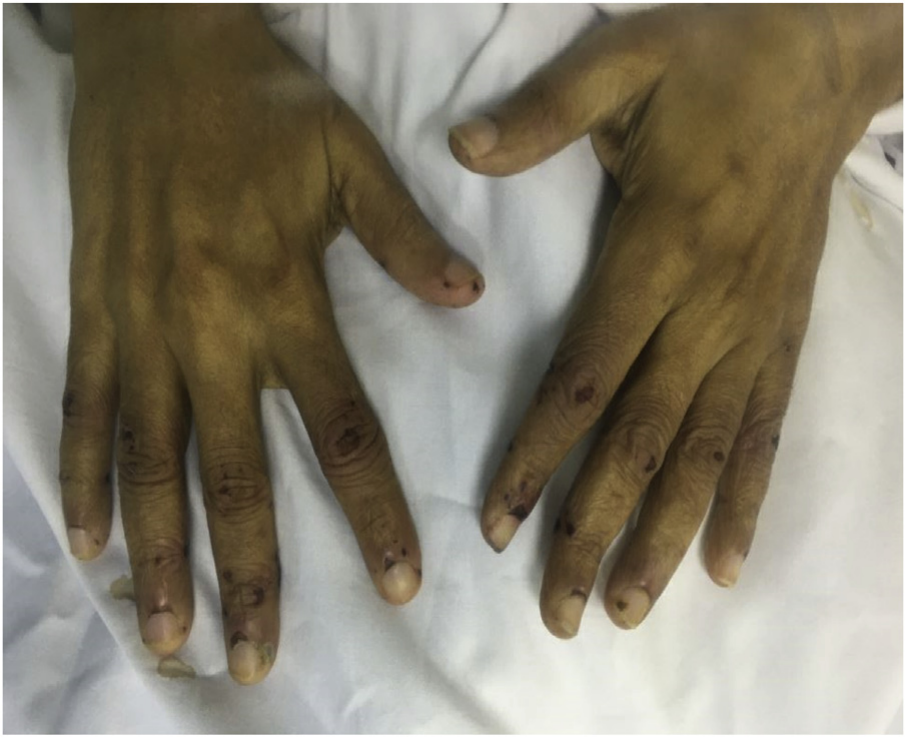
Skin lesions of the hands. Small areas of peripheral necrosis involving the fingers of both hands.

On physical examination the patient was tachycardic at 124 beats/minute but normo-tensive (124/76 mmHg), afebrile and maintaining normal oxygen saturation on room air. Central nervous system examination revealed a fully oriented individual with Glasgow Coma Scale (GCS) level 15, no sensory deficits throughout limbs, power of 4+/5 in the left upper limb and 5/5 in all other limbs. There were no positive cerebellar signs, no cranial nerve or sensory deficits and Babinski’s sign was negative on both lower limbs. Peripheral pulses were palpable and strong bilaterally in upper and lower limbs. Upon examination of the skin, the upper limbs were slightly erythematous and swollen in certain areas but non-tender. Additionally, present were multiple petechiae, palpable purpura and occasional non-tender necrotic ulcers distally on the fingers with no discoloration of the finger tips. Both lower limbs were also swollen and erythematous but non-tender. Multiple dark, necrotic, painless groups of ulcers and patches were present, with the largest lesion on the left foot and also spanning up the shins, as shown in Fig. 1, Fig. 2. The patient’s lower back revealed a petechial rash similar to the petechiae present on the upper limbs and there was no movement restriction in any areas containing the rash. Aside from this, examination of all other systems were unremarkable.

A CT scan with angiography of the brain was done that revealed no acute infarctions or bleeds and chest x-ray was normal. Initial investigations showed leukocytosis of 26.1(x10^3^/uL) with neutrophilia and a normal lymphocyte count. High inflammatory markers were noted with elevated C-reactive protein (CRP) levels of 305.5(mg/L) and procalcitonin (PCT) of 0.20(ng/mL) in addition to high D-Dimer, ferritin and lactate dehydrogenase (LDH). Random blood sugar level was 334(mmol/L) and glycated hemoglobin (Hba1c) was 13.2(mmol/mol).

Further lab studies unveiled multi-system involvement of the patient with abnormal cardiac enzymes, evidence of renal injury, and a deranged coagulation profile. Elevated prothrombin time (PT) of 17.4, international normalized ratio (INR) 1.34 and activated partial thromboplastin time (aPTT) 54.1 s were noted. Urinalysis with culture was normal, blood cultures were negative and liver function tests were normal. A full connective tissue disease and vasculitis work-up was ordered, which can be viewed in Table 1. Results were completely negative with the sole exception of a positive lupus anti-coagulant test. A naso-pharyngeal swab for SARS-CoV-2 was taken from the patient which eventually resulted as positive for the virus.

**Table 1:** Connective tissue disease and vasculitis work-up results.

INVESTIGATION	RESULT
Anti-Nuclear Antibody (ANA)	Negative
Anti-Double Stranded DNA Antibody	Negative
**Lupus Anti-coagulant**	**Positive**
Extractable Nuclear Antigen (ENA) Profile	Negative
Anti-Cardiolipin Antibodies	Negative
Complement - C3	Normal Levels
Complement - C4	Normal Levels
Rheumatoid Factor (IgG)	Negative
Anti-Phosphatidyl Serine (IgG)	Negative
Anti-Phosphatidyl Serine (IgM)	Negative
Cyclic Citrullinated Peptide (CCP) Antibodies	Negative
Anti-Beta-2 Glycoprotein 1 (IgG, IgM)	Negative
C – ANCA (Anti-Neutrophillic Cytoplasmic Antibody)	Negative
P - ANCA	Negative

The patient was admitted under the care of the Internal Medicine team as a case of transient ischemic attack (TIA) with concurrent COVID-19 infection for further management. During the stay, the patient was treated for his suspected TIA with Aspirin™ and statins in addition to other medications and the left arm weakness improved shortly after presentation. A skin biopsy was taken from the rash site with biopsy results detailing features suggestive of leukocytoclastic vasculitis. The Rheumatology team was consulted and based on lab investigations interpreted the patient having a case of secondary vasculitis due to COVID-19 infection.

**Fig. 3:**
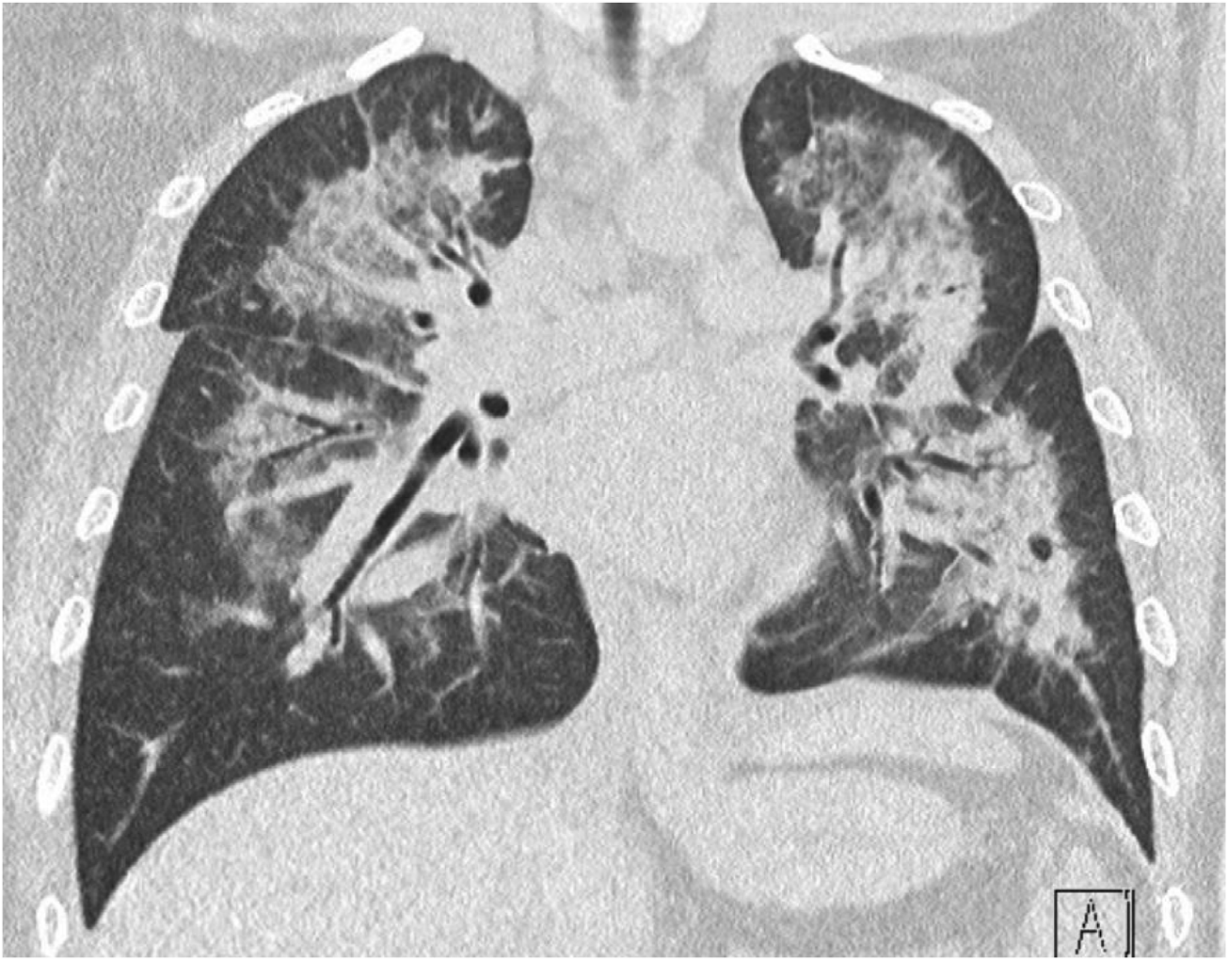
Computerized Tomography (CT) Scan of the Lungs. Consolidative patterns with air bronchograms involving both lung fields with scattered areas of patchy ground-glass opacities. Suggestive of an organizing pneumonia.

The Infectious Disease team began COVID-19 treatment with hydroxychloroquine. Unfortunately the patient still went on to deteriorate, requiring further oxygen therapy. Kaletra™ (lopinavir-ritonavir), Favipiravir, interferon-beta nebulizations, Tocilizumab and intra-venous (IV) immunoglobulin were further added to the treatment plan due to clinical worsening. With regards to COVID-19 treatment used, it should be noted that both hydroxychloroquine and Kaletra™ were initially temporarily approved in Dubai during the early stages of the pandemic. However, both these drugs are considered ineffective treatment for COVID-19 currently. Contrastingly, Favipiravir, Interferon beta, Tocilizumab and IV immunoglobulin are all still temporarily approved COVID-19 regimens in Dubai. A high resolution CT-chest was done soon after that revealed an organizing bilateral pneumonia as shown in Fig. 3. The Cardiology team were consulted for suspected heart failure based on the CXR and lower limb swelling. Trans-thoracic echocardiography (TTE) showed an ejection fraction of 30–35 %, myocardial infarction was ruled out and a diagnosis of systolic heart failure likely secondary to infection was made. Low dose Furosemide, IV Normal Saline infusion, Bisoprolol and Aspirin was started soon after. Treatment with Enapril was deferred in view of renal impairment.

The patient’s rash continued to worsen during his stay, even before starting the anti-heart failure therapy. Large necrotic patches with deep ulceration started forming in addition livedo reticularis of the upper limbs. At this point the patient started to complain of decreased sensation of bilateral lower limbs which was confirmed on examination, although peripheral pulses were still strong. A wound culture was positive for *Citrobacter* Freundii and Methicillin-Resistant Staphylococcus Aureus (MRSA) so Trimethoprim/Sulfamethoxazole and Ertapenem were started. Blood cultures and urine cultures were negative. Wound Management and General Surgery teams managed the patient’s wounds with surgical debridements and wound dressing.

**Fig. 4:**
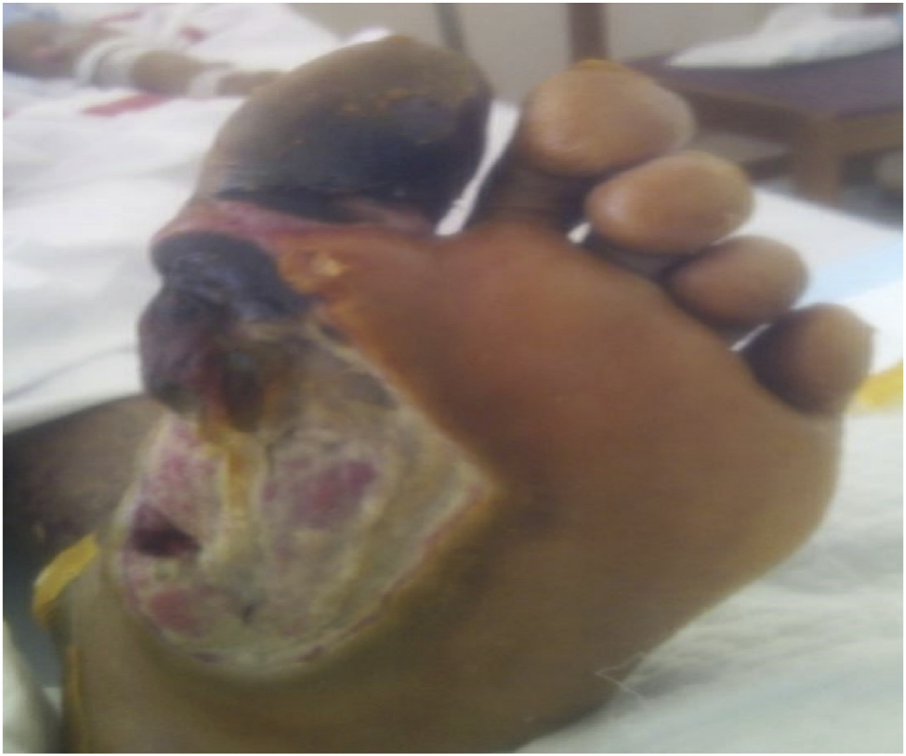
Left Plantar Foot Wound with Dry Gangrene. Left plantar foot wound post-multiple debridements due to prior large necrotic patch, measuring approximately 8 × 6 cms. Tendons visible within wound with underlying deep tissue. Prominent development of dry gangrene in the large toe involving the toe base.

**Fig. 5:**
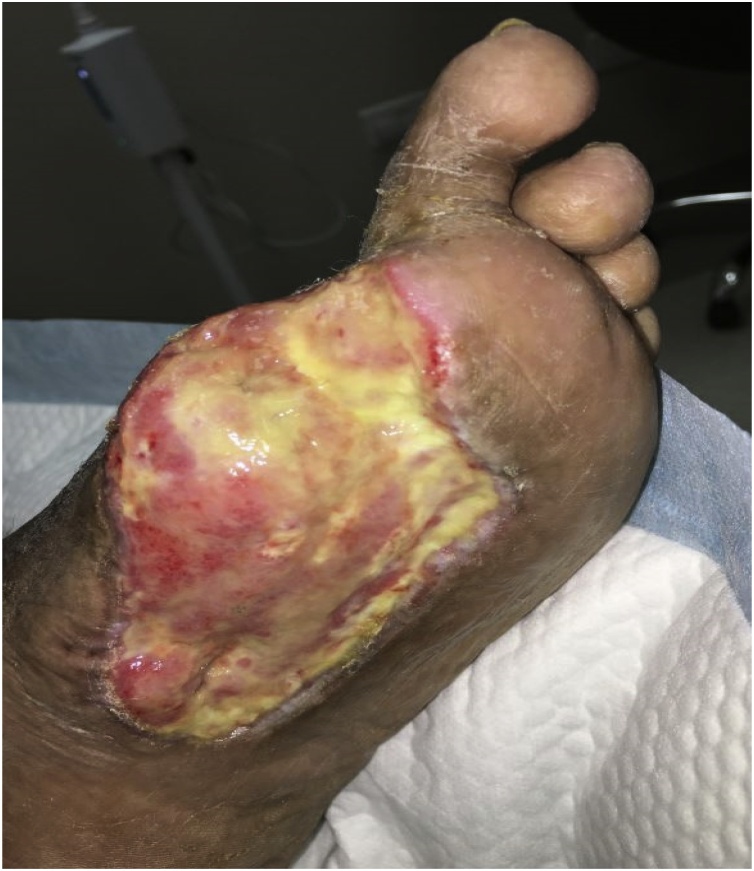
Healing Left Foot Wound Post-Amputation of Large Toe. Granulation tissue and slough can be seen within the wound with some serousanguinous exudate, dry wound edges and no signs of infection.

After more than a month of hospitalization, the patient improved and could maintain normal oxygen saturation on room air and did not experience any hypotensive episodes requiring vasopressors. Unfortunately, the lower limb wounds continued to worsen but X-ray of the lower limbs showed no evidence of osteomyelitis. Overtime the patient developed signs of ischemia of the left big toe which progressed to dry gangrene, as shown in Fig. 4. Due to this new occurrence, amputation of the left big toe was performed. Post-amputation and multiple debridements later, the patient’s foot became cleared of infection and well-healed, as seen in Fig. 5. The patient was finally discharged from the hospital in stable condition.

## Discussion

The Presentation and management of COVID-19 patients is still being questioned by researchers all around the world. This case is a clear example of uncommon multi-systemic manifestations associated with the novel coronavirus. A lingering issue during management of the patient was deducing a diagnosis that fit the entire clinical picture. It was imperative to consider the significance of the late lower limb decreased sensation experienced by the patient. This finding could have possibly pointed towards a diabetic foot with neuropathy, especially since the patient’s blood sugar levels on presentation were very uncontrolled. However, such an acute onset of bilateral neuropathy spanning up to the knees bilaterally seemed less likely to be the underlying cause. Especially when considering the entire patient picture and skin biopsy findings. It is possible however, that the poorly-controlled underlying diabetes along with the new vasculitic process combined to compromise distal blood supply, affecting the wound healing process. The impaired blood supply was not confirmed by a lower limb angiogram, as early-on lower limb distal pulses were palpable and strong. Once the necrosis had occurred it was clear that amputation would ultimately be needed, hence further vascular imaging was again not performed.

A wide-spectrum of manifestations have been illustrated since the virus’s emergence [3,4]. Rare but observed manifestations include increased thrombosis, myocardial dysfunction and various skin rashes. Arterial and venous thrombosis are postulated to occur due to coagulation derangements in infected patients caused by the release of inflammatory cytokines [5]. Production of anti-phospholipid antibodies is thought to be an additional reason, though less likely as they are often transient, especially the lupus anticoagulant [5]. These processes are relevant to our case, who’s TIA and other symptoms may have occured due to such hypercoagulable states. Unfortunately, we were unable to repeat the lupus anti-coagulant test in our patient to rule out a possible transient elevation or false positive result. The best management for these thrombotic events is still unclear [5].

Although this patient had multi-organ involvement, skin manifestations were evidently most problematic hence they were focused on during the case presentation. Leukocytoclastic vasculitis, also known as hypersensitivity vasculitis or cutaneous small vessel vasculitis (CSVV), is not commonly reported alongside COVID-19 [4]. Additionally, reported cases do not often experience such extents of necrosis and gangrene. Although successful treatment regimes are not often detailed, a reported case of COVID-19 associated vasculitis found success using oral steroids for treatment [6]. Skin manifestations overall have been noted in only a minority of patients, as evident in an Italian study finding only 20.4 % of their COVID-19 study population with cutaneous manifestations [7]. A large study done in China further emphasizes the rarity, with only 2 patients out of 1099 having skin findings [8]. Generally, the most common rashes experienced are maculo-papular rashes followed by papulo-vesicular rashes [4]. With regards to our presented case, seeing as C-ANCA (anti-neutrophil cytoplasmic antibody) and P-ANCA tests were negative, a secondary vasculitis such as CSVV due to COVID-19 infection seems the most likely explanation for the patient’s condition.

Histo-pathological skin biopsy findings of this vasculitis were discovered in this case, which included trans-epidermal necrosis, neutrophil-dense vasculitis and leukocytoclasis. The use of certain medications, viral infections and neoplastic processes are typically the most common triggers, with multiple theories behind the vasculitic process being suggested. One theory suggests that intra-vascular microthrombi formation hinders the bloody supply to the skin, causing necrotic patches [9]. A reported case documents antibodies as a possible cause, with its described patient having high titers of anti-cardiolipin IgM antibodies [10]. In the case we presented, anti-cardiolipin antibodies were negative while the lupus anti-coagulant was positive. The main theory however, suggests that the disease occurs due to activation of the complement and coagulation cascade as a result of endothelial blood vessel immune complex deposition [10]. A contrasting mechanism suggested involves the release of interleukin 6 (IL-6) in patients with lung disease [10]. The patient presented in this case did have pneumonia, however IL-6 levels were not measured. Complex neutrophilic processes may also occur in COVID-19 infections, leading to immune activation [11]. Regarding management of this hypersensitivity vasculitis, no clear guidelines are known to exist specific to the coronavirus. However, general management often depends on the etiology of the vasculitis and may include: treatment of underlying causes, glucocorticoids, colchicine, rest and possibly compression stockings [6].

The vast and unpredictable consequences of the novel coronavirus infection has been illustrated throughout this case. Previously, viruses were already known possible triggers of vasculitis, although not seen extremely often. This may similarly apply to the novel coronavirus, with potentially more grave consequences.

## Consent

Clear identifying patient details have been omitted in the case report. Details used will not reveal the patients identity.

## Funding

This research did not receive any specific grant from funding agencies in the public, commercial, or not-for-profit sectors.

## Author contribution

**Khalid Omar Alattar**: - Conceptualization, Resources, Writing (Original Draft), Writing (Review & Editing), Visualization.

**Farah Noaman Subhi**: Conceptualization, Resources, Writing (Original Draft), Project Administration.

**Ayesha Humaid Saif Alshamsi:** - Conceptualization, Resources, Writing (Original Draft), Writing (Review & Editing), Visualization, Project Administration.

**Nadereh Eisa**: - Conceptualization, Resources, Writing (Original Draft), Project Administration.

**Niaz Ahmed Shaikh:** Visualization, Supervision, Project Administration.

**Jehangir Afzal Mobushar**: Supervision, Project Administration.

**Asma Al Qasmi:** Supervision, Project Administration.

## Declaration of Competing Interest

The authors report no declarations of interest.

## References

[1] Cennimo D. (2020). Coronavirus disease 2019 (COVID-19): practice essentials, background, Route of transmission. https://emedicine.medscape.com/article/2500114-overview.

[2] Alramthan A., Aldaraji W. (2020). Two cases of COVID‐19 presenting with a clinical picture resembling chilblains: first report from the Middle East. Clin Exp Dermatol.

[3] Huang C., Wang Y., Li X. (2020). Clinical features of patients infected with 2019 novel coronavirus in Wuhan, China. Lancet.

[4] Casas C., Català A., Hernández G. (2020). Classification of the cutaneous manifestations of COVID‐19: a rapid prospective nationwide consensus study in Spain with 375 cases. Br J Dermatol.

[5] Beyrouti R., Adams M., Benjamin L. (2020). Characteristics of ischaemic stroke associated with COVID-19. J Neurol Neurosurg Psychiatr.

[6] Gota C., Calabrese L. (2013). Diagnosis and treatment of cutaneous leukocytoclastic vasculitis. Int J Clin Rheumtol.

[7] Recalcati S. (2020). Cutaneous manifestations in COVID‐19: a first perspective. J Eur Acad Dermatol Venereol.

[8] Guan W., Ni Z., Hu Y. (2020). Clinical Characteristics of Coronavirus Disease 2019 in China. N Engl J Med.

[9] Vleugels R. (2020). Hypersensitivity vasculitis: background, pathophysiology, etiology. https://emedicine.medscape.com/article/1083719-overview.

[10] Ibarguren A., Rodriguez M., Castanedo L. (2020). Cutaneous small vessel vasculitis secondary to COVID‐19 infection: a case report. J Eur Acad Dermatol Venereol.

[11] Sais G., Vidaller A., Jucglà A. (1998). Prognostic factors in Leukocytoclastic Vasculitis. Arch Dermatol.

